# Immunotherapy with IgY Antibodies toward Outer Membrane Protein F Protects Burned Mice against *Pseudomonas aeruginosa* Infection

**DOI:** 10.1155/2020/7840631

**Published:** 2020-05-29

**Authors:** Fatemeh Norouzi, Bahador Behrouz, Mahya Ranjbar, Seyed Latif Mousavi Gargari

**Affiliations:** ^1^Department of Biology, Faculty of Basic Science, Shahed University, Tehran, Iran; ^2^Department of Microbiology, Shahed University Faculty of Medical Sciences, Tehran, Iran

## Abstract

Burn patients with multidrug-resistant *Pseudomonas aeruginosa* infections commonly suffer from high morbidity and mortality, which present a major challenge to healthcare systems throughout the world. Outer membrane protein F (OprF), as a main outer membrane porin, is required for full virulence expression of *P. aeruginosa*. The aim of this study was to evaluate the protective efficacy of egg yolk-specific antibody (IgY) raised against recombinant OprF (r-OprF) protein in a murine burn model of infection. The hens were immunized with r-OprF, and anti-r-OprF IgY was purified using salt precipitation. Groups of mice were injected with different regimens of anti-OprF IgY or control IgY (C-IgY). Infections were caused by subcutaneous injection of *P. aeruginosa* strain PAO1 at the burn site. Mice were monitored for mortality for 5 days. The functional activity of anti-OprF IgY was determined by *in vitro* invasion assays. Immunotherapy with anti-OprF IgY resulted in a significant improvement in the survival of mice infected by *P. aeruginosa* from 25% to 87.5% compared with the C-IgY and PBS. The anti-OprF IgY decreased the invasion of *P. aeruginosa* PAO1 into the A549. Passive immunization with anti-OprF IgY led to an efficacious protection against *P. aeruginosa* burn infection in the burn model.

## 1. Introduction

*P. aeruginosa* has emerged as a formidable pathogen that contributes to fatal infections among burn patients to a great extent, primarily because they are notoriously resistant to a broad array of antimicrobial agents, which rapidly disseminate throughout the burn units worldwide [1–3]. Moreover, nosocomially acquired multidrug-resistant (MDR) strains of *P. aeruginosa* can spread systemically from the site of burn wound infection to distant organs, in part due to the immunosuppressive effects of burn trauma, in addition to the production of virulence factors that confer invasiveness, which may result in life-threatening systemic infections [4]. The global rising trend of morbidity of burn patients, combined with the dwindling choices of effective therapeutic options to treat MDR *P. aeruginosa* strains, has compelled researchers to investigate the merits of active as well as passive immunotherapy approaches in the treatment of severe burn wound infections.

Most clinical isolates of *P. aeruginosa* possess outer membrane protein F (OprF), encoded by the *oprF* gene which maintains the cell shape by anchoring the peptidoglycan to the outer membrane and is involved in host-pathogen interactions and also required for the expression of full virulence [5, 6]. For instance, studies have shown that non-OprF *P. aeruginosa* mutants have lower virulence in terms of impatience in ExoT and ExoS toxins through the type III secretion system (T3SS), Pseudomonas quinolone signal (PQS) synthesis, and production of the quorum-sensing-dependent virulence factors as well as biofilm development [7, 8]. Mounting evidence from several *in vitro* and *in vivo* studies supports the notion that OprF is surface exposed, is antigenically conserved, and could serve as a promising antigen for a vaccine against *P. aeruginosa* in various models of acute and chronic infections [9–13]. In addition, antibodies generated in response to OprF have been shown to exhibit potent antigen-binding, antibody-dependent, and complement-mediated opsonophagocytic killing activities against *P. aeruginosa* PAO1 [14], whose anti-OprF IgG activity level is correlated with the level of protection against *P. aeruginosa* in experimental animals and humans [15, 16]. Moreover, an adenovirus vector expressing OprF induces anti-OprF humoral and cellular immunity and provides protection against a lethal pulmonary challenge with *P. aeruginosa* [12].

Chicken egg yolk immunoglobulins (IgY) have been known as an extremely rich and economical source of polyclonal antibodies, which is not immunologically cross-reactive with the mammalian complement system and IgG [17]. Also, the high yield of specific antibodies along with simple and noninvasive collection method of IgY reveals a number of advantages over mammalian IgG antibodies to control infectious diseases [18]. IgY has been shown to prevent gastrointestinal [19] and influenza virus infections in both humans and animals without side effects [20, 21]. Oral immunotherapy with anti-*P. aeruginosa* IgY antibodies effectively declines chronic colonization of *P. aeruginosa* in CF patients [22, 23].

The present study evaluates the protective potential of anti-OprF IgY antibodies against *P. aeruginosa* in the burned mouse model of infection and determines the *in vitro* protective activity of elicited antibodies.

## 2. Materials and Methods

### 2.1. Bacterial Strains and Growth Media

*P. aeruginosa* PAO1 was used for the purification of the OprF protein and challenge. Luria-Bertani (LB) medium, trypticase soy agar (TSA), and tryptic soy broth (TSB; all from Merck, Germany) were used for routine culture of all bacterial strains.

### 2.2. Animals

Male 6–8-week-old BALB/C mice were purchased from the Royan Institute (Tehran, Iran). The 25 weeks old, shaver laying hens were purchased from a poultry farm (Alborz, Iran). All animal experiments were performed in compliance with the Animal Ethics Committee guidelines of Shahed University.

### 2.3. Preparation of Recombinant Protein

Recombinant OprF protein was purified as described previously. Briefly, the OprF gene (GenBank Accession No. NC_002516.2), previously cloned into the pET-28a vector, was transformed into *Escherichia coli* BL21. The recombinant gene construct was expressed with isopropyl b-D thiogalactoside (IPTG, 1 mM), and protein was affinity purified by a Ni-NTA agarose column under denaturing procedures (Qiagen, Hilden, Germany). The purified recombinant protein was confirmed by Western blotting with mouse anti-His tag monoclonal antibody.

### 2.4. Preparation of Anti-OprF IgY Antibodies

Two hens were immunized with 150 *μ*g of the r-OprF in complete Freund's adjuvant (1 : 1; Sigma-Aldrich, USA), which was administered intramuscularly and boosted 3 times with 150 *μ*g of this protein in incomplete Freund's adjuvant (1 : 1; Sigma-Aldrich), while control hen received adjuvant only at 2-week intervals. Two weeks after the last injection, the laid eggs were collected daily for 5 to 6 months and stored at 4°C. Isolation of anti-OprF IgY antibodies was performed as described previously [24]. The separated egg yolk was diluted 7 times with distilled water (pH 5) and incubated at -70°C overnight and then filtered with Whatman cellulose filter paper (Sigma-Aldrich). The filtrate was mixed with 8.8% (*w*/*v*) NaCl at pH 4 for 2 h and then centrifuged at 3,380 × g for 20 min. The pellet was dissolved in PBS, and final IgY was stored at -20°C. The purity of IgY was evaluated by 9% SDS-PAGE, followed by Coomassie Brilliant Blue G-250 staining. The total amount of IgY was quantitatively measured by the Bradford method. The reactivity of IgY antibodies raised against the r-OprF was analyzed by immunoblotting r-OprF. The r-OprF was transferred onto the nitrocellulose membrane from SDS-PAGE by wet transfer blotting apparatus. The membrane was washed by PBS and blocked with PBS containing 5% (*w*/*v*) skim milk overnight. The membrane was washed and incubated with 1 mg of anti-OprF IgY for 2 h in order to recognize r-OprF. 1 : 10000 diluted rabbit anti-chicken IgY conjugated with HRP (Sigma-Aldrich) was added as a secondary antibody and incubated at 37°C for 2 h, then washed with 0.05% Tween PBS (T-PBS). The paper was submerged in a solution containing 50 mM Tris (pH 7.8) and 0.6 mg/mL 3,3′-diaminobenzidine substrate (DAB). The reaction was terminated with distilled water after color development.

### 2.5. Evaluation of Anti-OprF IgY Titers

Antigen-specific IgY titers against whole-cell *P. aeruginosa* PAO1 as well as r-OprF were assessed by ELISA, as described previously [25]. Briefly, each ELISA plate well (Nunc, USA) was coated with 10^8^ CFU of *P. aeruginosa* PAO1 or 2.5 *μ*g r-OprF in 15 mM Na_2_CO_3_ and 35 mM NaHCO_3_ (pH 9.6), incubated overnight at 4°C, washed with 0.05% T-PBS, and blocked with PBS+5% skim milk. 100 *μ*L of 50 *μ*g/mL IgY antibodies was incubated in each well for 90 min at 37°C and washed three times with T-PBS, and then, 100 *μ*L of 1 : 1000-diluted HRP-conjugated rabbit anti-IgY antibody (HRP; Sigma-Aldrich) was added. After incubating for 1 h at 37°C, the plates were washed three times with T-PBS. Next, 100 *μ*L of TMB liquid substrate was added to each well. After color development for 20 min at room temperature, the reaction was stopped with 3 N H_2_SO_4_ and the absorbance at 450 nm (OD_450_) was measured.

### 2.6. Invasion Assay

To test the inhibitory activity of anti-OprF IgY antibodies on *P. aeruginosa* invasion to the A549 cell line, a gentamicin protection assay was performed, and we followed the methods of Ranjbar et al. [25]. Briefly, anti-OprF IgY antibodies were mixed with PAO1 strain and then added to confluent A549 cells seeded in a 24-well plate (Nunc). Gentamicin was then added to the plate and incubated for 1 h; then, cells were washed with PBS and lysed with 0.5% Triton X-100, and samples were serially diluted and grown on TSA plates (triplicates). Colony counting after 16 hours showed the number of PAO1 strain released from lysed cells.

### 2.7. Murine Burn Infection Model

The mice were burned and challenged as previously described by Neely et al. [26]. BALB/C mice (*n* = 56) were randomized into 7 groups. Briefly, 10-15% total body surface area (TBSA) burn wound was created using ethanol flame (0.5 mL ethanol). All mice received 0.3 mL of sterile saline intraperitoneal immediately after burning. Acetaminophen (0.25 mg/mL) was used post burn as an analgesic. The mice were challenged subcutaneously at the burn site with *P. aeruginosa* neutralized by preincubating with 0.1 and 10 mg of anti-OprF IgY antibodies. In other groups, *P. aeruginosa* were preincubated with 1 mg of anti-OprF IgY antibodies and then mice received intravenously 0.5 mg of anti-OprF IgY antibodies 12 h after infection. Moreover, in other groups, mice received 1 mg of anti-OprF IgY antibodies 2 h before infection and 0.5 mg of anti-OprF IgY antibodies 12 and 24 h after infection. In the IgY control group, mice were challenged subcutaneously with *P. aeruginosa* that were preincubated 1 h with 1 mg of control IgY (C-IgY). Survival without treatment was monitored in the PBS-treated group. The burn control group comprised untreated mice with burn wounds that were not infected. The survival rate of experimental mice was monitored twice daily up to 5 days, which were analyzed using the Mantel-Cox log-rank test [25].

### 2.8. Statistical Analysis

All statistical analyses were performed using GraphPad Prism 6 (GraphPad Software, Inc., USA). The data were analyzed by one-way analysis of variance with Tukey's multiple comparison tests. Survival analysis for different mouse groups was performed using the Kaplan Meier survival curve with the Mantel-Cox log-rank test [25]. All results were expressed as the mean ± standard deviation (SD). The *P* values less than 0.05 were considered statistically significant.

## 3. Results

### 3.1. Expression and Purification of r-OprF

**Figure 1:**
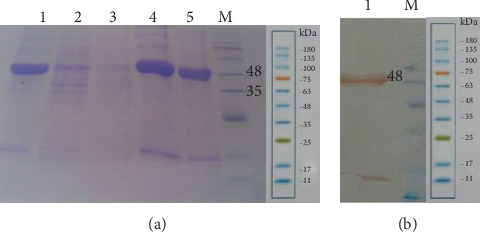
SDS-PAGE for detecting expressed and purified r-OprF. Lane M: low molecular weight protein size markers; Lane 1: precolumn lysate, Lane 2: flow through the matrix; Lane 3: washing with 20 mM imidazole; Lane 4: elution with 250 mM imidazole; Lane 5: purified r-OprF after dialysis (a). Western blotting results. Lane 1: r-OprF detected by monoclonal anti-His tag antibody; Lane M: low molecular weight protein size markers (b).

The protein expression of *E. coli* BL21 (DE3) carrying a recombinant vector was induced with IPTG (1 mM). Based on the SDS-PAGE, the expression product of r-OprF protein was approximately 48 kDa. The OprF was successfully purified by Ni–NTA affinity chromatography under denaturing procedures (Figure 1(a)). As illustrated in Figure 1(b), based on Western blot analysis, anti-His monoclonal antibody reacted specifically with a ∼48 kDa purified protein, corresponding to r-OprF.

### 3.2. The Reactivity and Specificity of IgY Antibodies Raised against OprF

**Figure 2:**
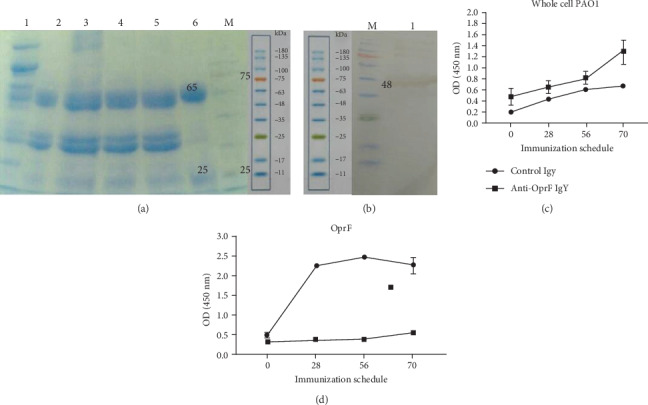
Precipitation and reactivity of anti-OprF IgY antibodies. SDS-PAGE of IgY precipitated with NaCl under acidic conditions. Lane 1: egg yolk; Lane 2: filtered diluted egg yolk, Lane 3: add NaCl; Lane 4: adjust pH 4; Lane 5: precipitated at room temperature for 2 h; Lane 6: purified IgY; Lane M: protein marker (a). R-OprF induced specific IgY binding to *P. aeruginosa* target antigen. IgY immunoreacted with r-OprF (∼48) protein (b). An indirect ELISA was used to determine the reactivity of IgY antibodies against r-OprF with *P. aeruginosa* strain PAO1 (c) and r-OprF (d). C-IgY served as negative controls. Values represent the mean of triplicate independent experiments ± standard deviation (SD).

The reactivity and specificity of IgY antibodies were evaluated using immunoblots of OprF. The IgY raised against r-OprF was precipitated by NaCl, and 50 mg of anti-OprF IgY was obtained per egg (Figure 2(a)). IgY antibodies from immunized egg yolk reacted with ∼48 kDa r-OprF protein (Figure 2(b)). The specificities of IgY antibodies raised against r-OprF were further verified using an indirect ELISA to analyze whole cell lysates as well as r-OprF. As shown in Figures 2(c) and 2(d), the IgY levels of r-OprF-immunized hen against whole live cells of *P. aeruginosa* PAO1 strain or recombinant protein were significantly (*P* < 0.01) higher than those of C-IgY over a period of time.

### 3.3. Anti-OprF IgY Antibodies Reduce P. aeruginosa Invasion

**Figure 3:**
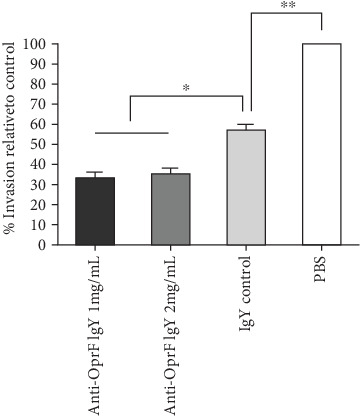
The inhibitory effects of anti-OprF IgY antibodies on the invasion of *P. aeruginosa* to A549 cells. PAO1 strain was incubated with different amounts of IgY antibodies (1 and 2 mg/mL). C-IgY and PBS served as controls. Values represent the mean of triplicate independent experiments ± SD. ^∗^*P* < 0.05 and ^∗∗^*P* < 0.01.

Anti-OprF IgY antibodies decrease the invasion to A549 cells by *P. aeruginosa*. The invasion efficiency of PAO1 in the presence of PBS was 100%. In contrast, in the presence of 1 and 2 mg/mL of anti-OprF IgY antibodies, invasion efficiencies of PAO1 were 33.72% and 35.08%, respectively, which were significantly higher than that of controls (*P* < 0.05, Figure 3). There was no significant difference between 1 and 2 mg/mL of anti-OprF IgY (*P* < 0.05). In the presence of C-IgY antibodies, the invasion efficiency of PAO1 was 58.02%, which was significantly higher than that of PBS (*P* < 0.05) (Figure 3).

### 3.4. Anti-OprF IgY Antibodies Increased the Survival of P. aeruginosa Infected Mice

**Figure 4:**
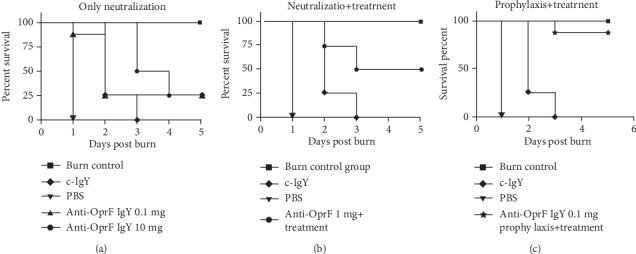
Protective effect of different anti-OprF IgY regimens on the survival of infected mice (*n* = 8) in comparison to control groups 5 days after subcutaneous inoculation of 10^8^ CFU *P. aeruginosa.* Mice received specific IgY as neutralized with bacteria (a), neutralized with bacteria treated with IgY after 12 h (b), and prophylaxis and treatment (c).

To assess the efficacy of anti-OprF IgY in rising protection against *P. aeruginosa* infection, we compared the survival rates of passively immunized mice with the anti-OprF IgY versus C-PBS- and C-IgY-infected mice (Table 1). The survival rates of infected mice with neutralized *P. aeruginosa* with both 0.1 and 10 mg of anti-OprF IgY were determined to be 25% (Figure 4(a)). Moreover, the survival rate of infected mice with neutralized *P. aeruginosa* with 1 mg of anti-OprF IgY and those that received 0.5 mg of anti-OprF IgY intravenously 12 h after infection was 50% (Table 1, Figure 4(b)). In addition, the survival rate of infected mice received 1 mg of anti-OprF IgY subcutaneously 2 h before infection as prophylaxis and treated with 0.5 mg of anti-OprF IgY intravenously 12 and 24 h after infection was 87.5%. None of the C-PBS and C-IgY mice survived *P. aeruginosa* wound infections (Table 1, Figure 4(c)). All noninfected burned mice survived.

## 4. Discussion

The foremost challenge in controlling *P. aeruginosa* burn wound infections is a limited success in antimicrobial therapy due to the emergence of MDR strains, which are highly resistant to virtually all available antimicrobial agents. Moreover, *P. aeruginosa* commonly evades the immune response and produces a wide array of virulence factors, which further damages the patient's organ systems. This further complicates patient treatment and leads to the exclusion of antibody-based immunotherapy. Although several *P. aeruginosa* antigens have been evaluated as possible vaccine candidates, OprF is known as a feasible target antigen because it is expressed and conserved antigenically in clinical isolates as well as having important functions during infection and providing protective antibody responses. In the current study, the burn wound mouse model has used to demonstrate that anti-OprF IgY antibodies afford protection against lethal *P. aeruginosa* infections. Our result showed that burned challenged mice were protected and their survival rates were higher than control groups. The results of the burned mouse model indicated that prophylaxis of *P. aeruginosa* infection by anti-OprF IgY antibodies and intravenous injection of anti-OprF IgY antibodies as treatment led to an increase of 87.5% in the survival rate of mice compared to the control group. Our findings are consistent with Matthews-Greer and Gilleland [5], who showed active immunization with isolated OprF from cell envelope led to an increase of 83% in the survival rate of burned mice after challenge with *P. aeruginosa*. Additionally, Worgall et al. demonstrated that active immunization with adenovirus expressing *P. aeruginosa* OprF increased the survival rate of infected mice in acute pneumonia model to 80% [12]. This also accords with our earlier observation where preincubation of P. aeruginosa with anti PcrV IgY enhanced the survival rate of burned mice to 33% as our 25% in the same preincubated group [25]. It is crucial to consider this statement, especially in the era of increasing number of drug-resistant bacteria and predominant MDR-*P. aeruginosa* strains in numerous hospitals, principally in burn units. In addition, the complex issue of successfully eradicating virulent and highly resistant bacterial strains within burn patients is further exacerbated with the issue of dwindling number of newly approved antimicrobial agents against such strains. Fortunately, mounting evidence has indicated that immunotherapy is a promising treatment option that holds potential as an independent therapeutic strategy, alone or in combination with antimicrobial therapy [27, 28]. It seems rational to consider that antibody-based immunotherapy prevents MDR-*P. aeruginosa* burden among the burn patient in whom infection is being established, which ultimately causes high morbidity and mortality. However, inhibition of *P. aeruginosa* virulence factor OprF by IgY antibodies shows a specific antibacterial effect without triggering the development of resistant strains.

In this study, we found that bacterial invasion to A549 cells was inhibited by anti-OprF IgY antibodies, which indicated a key role in reducing the local and systemic distribution of *P. aeruginosa*. It was previously found that high hydrophobicity of anti-OprF IgY antibodies aggregates bacteria, therefore facilitating clearance by the host immune cells [29]. The findings of the current study are consistent with that of our previous report of 25% invasion of *P. aeruginosa* to A549 cells in the presence of 1 mg anti-PcrV IgY [25]. The observed substantial clinical efficacy of IgY immunotherapy may be associated with interference interactions between pathogen and host epithelial cells [30, 31]. Furthermore, anti-OprF IgGs exhibit potent antibody-dependent complement-mediated killing of the *P. aeruginosa* strain PAO1 [14], and the levels of antibodies correlate with the levels of protection against *P. aeruginosa* in burned mice [16]. In addition, it was suggested that IgY antibodies have inhibitory effects on bacterial pathogenesis and can be considered an adjunct therapy to improve antibiotic action. Thus, anti-OprF IgY antibodies showed a great activity against *P. aeruginosa* and interfered with the *P. aeruginosa* virulence factor to inhibit cell invasion. The moderate inhibitory activity of C-IgY having a nonsignificant reduction in the invasion of *P. aeruginosa* and improvement in the survival of infected mice compared to anti-OprF IgY could be due to the exposition of chickens with *P. aeruginosa*, which is a ubiquitous environmental bacterium and also polyclonal nature of IgY. These findings are consistent with previous studies [25, 29, 32–37].

## 5. Conclusion

In conclusion, these results offer evidence that anti-OprF IgY antibodies can confer protection against burn wound infection caused by *P. aeruginosa* through the inhibition of bacterial invasion to host cells and tissues. Our data show that *P. aeruginosa*-infected treated mice are protected against burn wound sepsis, further supporting the conclusion that IgY against OprF provides approaches to develop a protective treatment. Supposedly, anti-OprF IgY antibodies may be used in combination with antibiotic therapies as an adjunct approach to prevent *P. aeruginosa* infections.

## Figures and Tables

**Table 1:** The effect of different regimens of anti-OprF IgY antibodies on the survival of *P. aeruginosa*-infected mice (*n* = 8). Survival was assessed in infected mice with burn wounds days after subcutaneous injection of *P. aeruginosa.*

Group	Challenge	Intravenous treatment	No. of dead mice/total no. of mice on day						Survival (%)
			1	2	3	4	5		
I	Neutralized *P. aeruginosa* with 0.1 mg of anti-OprF IgY	—	1/8	6/8	6/8	6/8	6/8^∗^		25
II	Neutralized *P. aeruginosa* with 10 mg of anti-OprF IgY	—	0/8	0/8	4/8	6/8	6/8^∗^		25
III	Neutralized *P. aeruginosa* with 1 mg of anti-OprF IgY	0.5 mg of anti-OprF IgY (12 h after infection)	0/8	2/8	4/8	4/8	4/8^∗∗^		50
IV	Prophylaxis 1 mg of anti-OprF IgY 2 h before infection with *P. aeruginosa*	0.5 mg of anti-OprF IgY (12 h and 24 h after infection)	0/8	0/8	1/8	1/8	1/8^∗∗^		87.5
V	Neutralized *P. aeruginosa* with 1 mg of control IgY	—	0/8	6/8	8/8				0
VI	*P. aeruginosa*	—	8/8						0
VII	—	—	0/8	0/8	0/8	0/8	0/8^∗∗^		100

^∗^ *P* < 0.05 and ^∗∗^*P* < 0.01 (Mantel-Cox log-rank test).

## Data Availability

No data were used to support this study.
